# Broadening the Clinical Spectrum of Axonal Hereditary Neuropathies: A Comparative Case Study on *DNAJB2‐* and *HINT1‐*Related Disease

**DOI:** 10.1111/jns.70100

**Published:** 2026-01-19

**Authors:** Bogdan Bjelica, Corinna Hendrich, Sandra von Hardenberg, Milica Vukojevic, Sonja Körner, Thomas Gschwendtberger, Aiden Haghikia, Stojan Peric, Susanne Petri

**Affiliations:** ^1^ Department of Neurology Hannover Medical School Hannover Germany; ^2^ PRACTIS Clinician Scientist Program, Dean's Office for Academic Career Development, Hannover Medical School Hannover Germany; ^3^ Department of Human Genetics Hannover Medical School Hannover Germany; ^4^ Neurology Clinic, University Clinical Center of Serbia, Faculty of Medicine, University of Belgrade Belgrade Serbia

**Keywords:** DNAJB2 neuropathy, hereditary axonal neuropathy, HINT1 neuropathy, non‐motor symptoms, quality of life

## Abstract

**Background and Aims:**

Differentiating hereditary axonal polyneuropathies caused by distinct gene variants remains a clinical challenge. This comparative case study of *DNAJB2*‐ and *HINT1*‐related neuropathies aimed to broaden the phenotypic spectrum associated with these genes and to explore non‐motor symptoms and quality of life (QoL) in affected individuals.

**Methods:**

Six patients carrying two novel *DNAJB2* variants and six age‐matched patients with *HINT1* variants underwent detailed clinical and electrophysiological characterization. Motor function was assessed longitudinally using the Medical Research Council (MRC) scale. Non‐motor symptoms (neuropathic pain, autonomic dysfunction, depression, fatigue, restless legs syndrome) and QoL were evaluated with patient‐reported outcomes and compared to four healthy controls (HC).

**Results:**

Both patient groups exhibited a CMT2 phenotype. Nerve conduction studies revealed a length‐dependent axonal predominantly motor but not pure motor neuropathy in most of the patients. Disease onset tended to occur later in patients with *DNAJB2* variants, who yet developed more severe neuropathy. The spectrum of additional clinical features differed between the two groups. All patients with *DNAJB2* variants fulfilled criteria for depression, compared with one with a *HINT1* variant. Significant fatigue was present in the majority of both groups, while restless legs syndrome was observed in four patients with a *DNAJB2* variant but in none with a *HINT1*. QoL was significantly reduced in *DNAJB2* versus HC, with no difference in QoL between patients with *DNAJB2* and *HINT1* variants.

**Interpretation:**

This study expands the clinical spectrum of *DNAJB2*‐ and *HINT1*‐related neuropathies, highlighting distinct non‐motor features and their impact on QoL, and providing the first direct comparison of these two rare axonal disorders.

## 1 Introduction

Hereditary polyneuropathies are a clinically and genetically heterogeneous group of disorders of the peripheral nervous system caused by pathogenic variants in more than 100 genes [1]. The most common form is Charcot–Marie–Tooth disease (CMT), which is further classified into demyelinating CMT (CMT1 and CMT4), axonal (CMT2), and intermediate (CMTX1) forms [2]. Distal hereditary motor neuropathies (dHMN) are a related group of hereditary neuropathies characterized by slow and progressive degeneration of the distal lower motor neurons, typically without significant sensory involvement. There is a clinical overlap between dHMN and CMT, as well as other degenerative diseases, such as spinal muscular atrophy (SMA), amyotrophic lateral sclerosis (ALS), hereditary spastic paraplegia (HSP), and myopathies [3].

Interpreting the pathogenicity of novel variants and the genotype–phenotype correlation remains a significant challenge in hereditary neuropathies, especially in axonal forms [3]. Loss‐of‐function variants in the histidine triad nucleotide‐binding protein 1 (*HINT1*) and Dnaj homolog subfamily B member 2 (*DNAJB2*) gene are considered rare causes of autosomal recessive axonal neuropathies, often associated with additional clinical features besides distal muscle weakness [4, 5]. So far, less than 20 recessive *DNAJB2* variants have been identified as disease causing [6], which may present either as dHMN or CMT2. Symptom onset in patients with *DNAJB2*‐related neuropathy typically occurs in the second decade of life and manifests as a progressive, primarily distal muscle weakness [7, 8, 9, 10, 11]. Additional features, including bulbar and respiratory symptoms in advanced stages [10], early‐onset parkinsonism [10, 11, 12], behavioral changes [10], and cerebellar ataxia [12] have been reported. Zimoń et al. reported in 2012 the first cases of hereditary polyneuropathy linked to eight loss‐of‐function recessive variants in the *HINT1* gene [4]. Similar to *DNAJB2*, patients with *HINT1*‐related neuropathy typically present with a progressive CMT2 or dHMN beginning in the first or second decade of life, leading to lower limb weakness and gait disturbances [13]. An additional clinical hallmark of HINT1 deficiency is neuromyotonia, a peripheral nerve hyperexcitability characterized by spontaneous muscle activity at rest and delayed muscle relaxation following voluntary contraction [13]. HINT1 appears to be one of the most common CMT2 in some countries [14]. Little is known about the presence of non‐motor symptoms (such as autonomic dysfunction, fatigue, restless legs syndrome [RLS], neuropathic pain, depression), as well as about overall quality of life (QoL) in patients with *DNAJB2*‐ and *HINT1*‐related neuropathies.

Here we report six patients from two families with novel homozygous *DNAJB2* variants and six patients from five families with homozygous *HINT1* variants. This study aimed to expand the clinical phenotype associated with these genes, additionally providing new insights into non‐motor symptoms and QoL in affected individuals.

## 2 Materials and Methods

### 2.1 Patient Recruitment and Data Collection

The Ethical Boards of included centers approved the study, and all participants gave their written informed consent to participate. In this observational descriptive study, we evaluated six patients from two families with homozygous variants in the *DNAJB2* gene, diagnosed and treated at the Department of Neurology of the Hannover Medical School. Six additional age‐matched patients from five families with a homozygous variant in the *HINT1* gene were diagnosed and treated at the Neurology Clinic, University Clinical Center of Serbia and evaluated as a disease comparison group. Sociodemographic and clinical data, encompassing sex, age at examination, age at onset, first symptom, ambulatory status (normal; abnormal—no aids required; abnormal—walking aids used; wheelchair outdoors/long trips; mostly confined to a wheelchair), tendon reflexes, sensory nervous system affection (joint position sense; vibration (ankle); pain/touch), presence of additional features (neuromyotonia, pes cavus, scoliosis, tremor, bradykinesia, spasticity, sensory ataxia, deafness, cerebellar dysfunction, bulbar dysfunction, diaphragmatic dysfunction, ulcerations, nystagmus, abnormal pupillary reaction, and other), motor function and electrophysiology results were collected at the time of testing. All patients underwent nerve conduction studies (NCS) according to the standard operating procedures of the respective institutions. As a healthy comparison group, four age‐matched healthy controls (HC) were included. Creatine kinase (CK) levels were measured using standardized methods at the central laboratories of both centers.

### 2.2 Genetic Analysis

Sample preparation, whole exome sequencing (WES), whole genome sequencing (WGS), and sequence variant analysis of patients with *DNAJB2* variants were performed as previously described [15]. Reads from WES and WGS were aligned to the human reference genome GRCh37/hg19. We analyzed genes that are associated with the respective phenotype and excluded intronic variants. The interpretation of sequence variants was based on the standards and guidelines of the American College of Medical Genetics and Genomics (ACMG) [16]. The variants in the *DNAJB2* gene refer to the following transcript: ENST00000336576.5.

### 2.3 Assessment of Motor Function, Disease Severity and Functional Disability

Evaluation of muscle strength was conducted based on the Medical Research Council (MRC) scale, ranging from zero to five, where zero signifies no muscle movement and five indicates normal muscle strength. Strength of the following muscles was included in the MRC sum score (MRC‐SS): shoulder abductors, elbow flexors, wrist extensors, hip flexors, knee extensors, and foot dorsiflexors [17]. Disease severity was determined using the Charcot–Marie–Tooth Neuropathy Score (CMTNS) [18]. Severity was categorized as mild if the CMTNS score was ≤ 10, moderate if between 11 and 20, and severe if > 20. Assessment of functional disability was done using the Overall Neuropathy Limitation Scale (ONLS) [19], which represents a tool for evaluation of functional disability of limbs and the ability to carry out daily activities, where higher scores mean higher functional disability.

### 2.4 Assessment of Non‐Motor Symptoms

Non‐motor symptoms were assessed in five patients with variants in *DNAJB2*, as one patient (patient 6) declined to participate in this part of the study. The results were then compared to six patients with *HINT1* variants and four HC.

RLS diagnosis was established according to the International Restless Legs Syndrome Study Group criteria [20]. Patients were screened to ensure the absence of significant conditions that could influence RLS presence, such as renal or inflammatory disorders, iron deficiency, or diabetes mellitus. RLS symptom severity was assessed utilizing the International Restless Legs Syndrome Severity Scale (IRLS‐SS) questionnaire. This questionnaire comprises 10 questions, each rated from zero to four, resulting in a total score range of zero to 40, where higher scores indicate more severe RLS symptoms. Severity was categorized as follows: moderate (IRLS‐SS score 10–20), severe (IRLS‐SS score 21–30), and very severe (IRLS‐SS score 31–40) [21].

Presence and severity of fatigue were evaluated using the Krupp's Fatigue Severity Scale (FSS). This questionnaire comprises nine self‐reported questions measuring the intensity of fatigue experienced over the past week. Higher scores on the scale indicate more severe fatigue. Presence of fatigue was defined as a total score of ≥ 36 [22].

Neuropathic pain was evaluated in accordance with the International Association for the Study of Pain (IASP) criteria [23]. This assessment was further substantiated using the painDETECT questionnaire (PD‐Q). PD‐Q is a self‐report assessment tool, consisting of nine items (seven descriptors of pain and two items related to spatial and temporal pain characteristics). A PD‐Q scale score ≥ 19 signified a definitive presence of neuropathic pain [24].

Symptoms of autonomic dysfunction were assessed using the Scale for Outcomes in Parkinson's disease for Autonomic symptoms (SCOPA‐AUT), a comprehensive self‐reported questionnaire containing 25 items. This tool covers various domains of autonomic function including gastrointestinal (seven items), urinary (six items), cardiovascular (three items), thermoregulatory (four items), pupillomotor (one item), and sexual function (two items). Each item evaluates the frequency of autonomic symptoms over the past month (except for syncope—six months) on a four‐point scale, ranging from zero (never) to three (often). The total SCOPA‐AUT score ranges from zero to 69, with higher scores indicating greater impairment in autonomic function [25]. The authors obtained permission for the use of the SCOPA‐AUT, as approved by the Movement Disorder Society.

Depression was assessed using the Beck Depression Inventory (BDI), with higher scores indicating a higher level of depression. Scores of 11 or higher were considered indicative of depression [26].

The health‐related QoL of patients was evaluated using the 36‐Item Short Form Survey (SF‐36) questionnaire [27]. This questionnaire encompasses eight general health domains: physical functioning (PF), role physical (RP), bodily pain (BP), general health (GH), vitality (VT), social functioning (SF), role emotional (RE), and mental health (MH). From these domains, two main scores are derived: the physical composite score (PCS) and the mental composite score (MCS), in addition to the total SF‐36 score. Each domain is scored from 0 to 100, with higher scores indicating a better QoL.

### 2.5 Statistical Analysis

All statistical analyses and graphs were performed using IBM SPSS (version 28, Chicago IL, USA) and GraphPad Prism (version 10.6.1, GraphPad Software Inc., San Diego, Californian, USA). Data were expressed as box plots with min to max (all data points shown) and significance level was set as *p* < 0.05. Results were compared by one‐way or two‐way ANOVA with Tukey's post‐test. Correlations were determined with Spearman's rank (correlation) coefficient.

To assess longitudinal changes in motor function in patients with *DNAJB2* variants, we fitted a linear mixed‐effects model with MRC‐SS score as the dependent variable. Time (in years since the first examination) was entered as a fixed effect. A random intercept for each patient was included to account for between‐subject variability, and within‐subject correlations across repeated measurements were modeled using a first‐order autoregressive covariance structure. Model parameters were estimated using maximum likelihood, and denominator degrees of freedom were calculated using Satterthwaite's approximation.

## 3 Results

### 3.1 Patients With *DNAJB2* ‐Related Hereditary Neuropathy

Five patients from a consanguineous Syrian family were found to carry a pathogenic homozygous variant (c.446‐1G>A) in the *DNAJB2* gene. Additionally, one patient from a consanguineous Iranian family carried a homozygous variant of uncertain significance (VUS) in the same gene (c.99C>G). The sociodemographic and clinical data of these patients are summarized in Table 1.

**TABLE 1:** Sociodemographic and clinical features of patients with *DNAJB2*‐related hereditary neuropathy.

Characteristics	Patients					
	Patient 1	Patient 2	Patient 3	Patient 4	Patient 5	Patient 6
	Family 1, I.1	Family 1, I.2	Family 1, I.3	Family 1, I.4	Family 1, I.5	Family 2, I.1^a^
Genetic variant	c.446‐1G>A p.? homozygous	c.446‐1G>A p.? homozygous	c.446‐1G>A p.? homozygous	c.446‐1G>A p.? homozygous	c.446‐1G>A p.? homozygous	c.99C>G p.(Asp33Glu) homozygous
Origin	Syrian	Syrian	Syrian	Syrian	Syrian	Iranian
Age at examination (years)	22	40	44	30	26	37
Sex	Female	Male	Male	Female	Male	Male
Age at onset (years)	17	20	21	20	18	21
Phenotype	CMT2	CMT2	CMT2	CMT2	CMT2	CMT2
First symptom	Foot weakness	Abnormal gait	Foot weakness	Abnormal gait	Foot weakness	Foot weakness; leg cramps
Upper limbs weakness						
Proximal (shoulder abductors)	5/5	5/5	5/5	5/5	5/5	4/5
Distal (wrist extensors)	5/5	5/5	4+/5	4+/5	4/5	4−/5
Lower limbs weakness						
Proximal (hip flexors)	4/5	4−/5	4−/5	4+/5	4/5	3/5
Distal (foot dorsiflexors)	2/5	1/5	0/5	2/5	1/5	0/5
Ambulation						
Normal						
Abnormal – no aids required	X			X	X	
Abnormal – walking aids used		X	X			
Wheelchair outdoors/long trips						
Mostly confined to a wheelchair						X
Tendon reflexes						
Biceps	Diminished	Diminished	Diminished	Diminished	Diminished	Absent
Triceps	Diminished	Diminished	Diminished	Diminished	Diminished	Absent
Knee	Diminished	Absent	Absent	Diminished	Diminished	Absent
Ankle	Absent	Absent	Absent	Absent	Absent	Absent
Sensory						
Joint position sense	Normal	Normal	Reduced up to the wrist/ankle	Reduced up to the wrist/ankle	Reduced up to the wrist/ankle	Reduced up to the wrist/ankle
Vibration	Reduced at elbow/knee	Reduced above elbow/knee	Reduced above elbow/knee	Reduced at elbow/knee	Reduced above elbow/knee	Reduced at elbow/knee
Pain/touch	Reduced in fingers/toes	Reduced above elbow/knee	Reduced above elbow/knee	Reduced up to and may include elbow/knee	Reduced above elbow/knee	Reduced up to and may include elbow/knee
Nerve conduction studies	Axonal motor neuropathy	Axonal predominantly motor neuropathy	Axonal motor neuropathy	Axonal motor and sensory neuropathy	Axonal motor neuropathy	Axonal motor and sensory neuropathy
Additional features						
Neuromyotonia	No	No	No	No	No	No
Pes cavus	Yes	Yes	Yes	Yes	Yes	Yes
Scoliosis	No	Yes	Yes	Yes	Yes	Yes
Tremor	No	No	No	No	Yes^b^	Yes^b^
Bradykinesis	No	No	No	No	No	No
Spasticity	No	No	No	No	No	No
Sensory ataxia	No	No	No	No	Yes	No
Deafness	No	No	Yes^d^	No	No	No
Cerebellar dysfunction	No	No	No	No	No	No
Bulbar dysfunction	No	Yes^c^	Yes^c^	No	No	No
Diaphragmatic dysfunction	No	No	No	No	No	No
Ulcerations	No	No	No	No	No	No
Nystagmus	No	No	No	No	No	No
Abnormal pupillary reaction	No	No	No	No	No	No
Other	No	No	No	No	No	No
CK level	285 U/L	679 U/L	305 U/L	663 U/L	1158 U/L	666 U/L
CMTNS	10	19	21	13	19	24
Severity	Mild	Moderate	Severe	Moderate	Moderate	Severe
ONLS						
Arms	0	0	2	0	2	2
Legs	2	3	3	2	2	5
Total	2	3	5	2	4	7

*Note:* “p.?” indicates that an effect on the protein level is expected, but that it is not possible to give a reliable prediction of the consequences.

Abbreviations: CK, creatine kinese; CMT2, Charcot–Marie‐tooth neuropathy type 2; CMTES, Charcot–Marie‐tooth examination score; CMTNS, Charcot–Marie‐tooth neuropathy score; ONLS, overall neuropathy limitations scale.

^a^ This patient had a variant of uncertain significance (VUS).

^b^ Mild rest and postural tremor.

^c^ Mild dysphagia.

^d^ Mild deafness.

Most patients developed symptoms during late adolescence, with initial manifestation occurring in the lower limbs in all cases. None of the patients had a normal gait pattern; one patient (patient 6) was wheelchair‐bound. Among the five patients with the *DNAJB2* c.446‐1G>A homozygous variant, four showed an axonal motor neuropathy on NCS, while one displayed an axonal motor and sensory neuropathy. Nevertheless, all patients demonstrated clinically diminished or absent function in at least one sensory modality (Table 1), consistent with a CMT2 phenotype. The patient with the homozygous VUS also exhibited an axonal motor and sensory neuropathy on NCS and a CMT2 phenotype (Table 2). Based on CMTNS, two patients had severe neuropathy (P3, P6), three had moderate neuropathy (P2, P4, P5), and one had borderline mild neuropathy (P1).

**TABLE 2:** Nerve conduction studies in patients with *DNAJB2*‐related hereditary neuropathy.

	Motor nerves								Sensory nerves					
	Median nerve		Ulnar nerve		Tibial nerve		Peroneal nerve		Median nerve		Ulnar nerve		Sural nerve	
	NCV, m/s	CMAP, mV	NCV, m/s	CMAP, mV	NCV, m/s	CMAP, mV	NCV, m/s	CMAP, mV	NCV, m/s	SNAP, μV	NCV, m/s	SNAP, μV	NCV, m/s	SNAP, μV
Patient 1	59.0	11.5	57.0	9.5	NA	NR	28.0	0.8	56.0	16.4	67.0	14.3	43.0	19.5
Patient 2	48.0	4.9	n.d.	n.d.	n.d.	n.d.	NA	NR	48.0	15.2	n.d.	n.d.	38.0	4.1
Patient 3	62.0	7.8	64.0	14.0	NA	NR	NA	NR	53.0	7.4	57.0	6.1	40.0	11.8
Patient 4	55.0	9.0	61.0	10.3	28.0	0.3	39.0	0.2	56.0	9.7	56.0	19.3	39.0	2.4
Patient 5	47.0	8.9	n.d.	n.d.	49.0	0.2	39.0	1.7	n.d.	n.d.	n.d.	n.d.	43.0	5.3
Patient 6	50.0	4.7	51.0	4.9	NA	NR	NA	NR	56.0	2.4	52.0	1.2	57.0	2.7

Abbreviations: CMAP, compound muscle action potential; NA, not applicable; NCV, nerve conduction velocity; n.d., not done; NR, no response to stimulation; SNAP, sensory nerve action potential.

**FIGURE 1:**
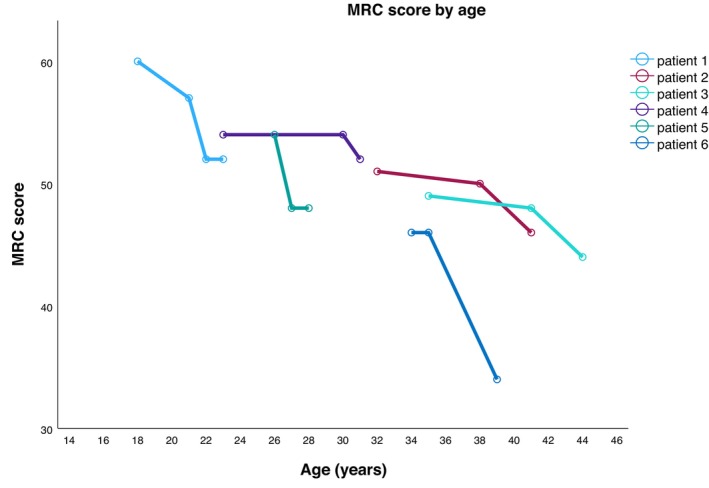
Longitudinal data on motor function in patients with *DNAJB2*‐related hereditary neuropathy. MRC, medical research council sum score.

All six patients had pes cavus; five had scoliosis (P2‐6). Mild postural tremor was observed in two patients (P5, P6). Bulbar dysfunction with mild dysphagia was present in two patients (P2, P3). Sensory ataxia and mild hearing loss were each observed in single cases (P5, P3, respectively). All patients showed progressive loss of motor function during follow‐up (range: 3–10 years) (Figure 1). Over the observation period, motor function declined on average by 0.78 points per year (on MRC‐SS scale) (B = −0.780, SE = 0.273, t(5.94) = −2.86, *p* = 0.029).

### 3.2 Patients With *HINT1* ‐Related Hereditary Neuropathy

Six patients from five non‐consanguineous Serbian families were found to carry a pathogenic homozygous variant (c.110G>C) in the *HINT1* gene. The sociodemographic and clinical data of these patients are summarized in Table 3. Most patients developed symptoms during childhood, while one patient experienced symptom onset in early adolescence. All patients exhibited the first symptom in the lower limbs. None of the patients had a normal gait pattern; no patient was wheelchair‐bound. All patients exhibited a CMT2 phenotype. Based on CMTNS, two patients had mild neuropathy (P1, P3) and four patients exhibited moderate neuropathy (P2, P4, P5, P6).

**TABLE 3:** Sociodemographic and clinical features of patients with *HINT1*‐related hereditary neuropathy.

Characteristics	Patients					
	Patient 1	Patient 2	Patient 3	Patient 4	Patient 5	Patient 6
	Family 1, I.1	Family 2, I.1	Family 3, I.1	Family 4, I.1	Family 5, I.1	Family 5, I.2
Genetic variant	c.110G>C p.Arg37Pro homozygous	c.110G>Cp.Arg37Pro homozygous	c.110G>C p.Arg37Pro homozygous	c.110G>Cp.Arg37Pro homozygous	c.110G>C p.Arg37Pro homozygous	c.110G>C p.Arg37Pro homozygous
Origin	Serbian	Serbian	Serbian	Serbian	Serbian	Serbian
Age at examination (years)	50	54	26	21	36	34
Sex	Female	Male	Male	Male	Female	Female
Age at onset (years)	3	10	1	13	7	7
Phenotype	CMT2	CMT2	CMT2	CMT2	CMT2	CMT2
First symptom	Foot weakness	Abnormal gate and foot weakness	Contracture of the tendon, foot weakness	Foot weakness and scoliosis	Foot weakness and scoliosis	Abnormal gate and foot weakness
Upper limbs weakness						
Proximal (shoulder abductors)	5/5	5/5	5/5	5/5	4/5	4/5
Distal (wrist extensors)	4/5	4/5	5/5	4/5	4/5	4/5
Lower limbs weakness						
Proximal (hip flexors)	5/5	4/5	5/5	5/5	4/5	4/5
Distal (foot dorsiflexors)	0/5	1/5	0/5	2/5	3/5	3/5
Ambulation						
Normal						
Abnormal – no aids required	X			X	X	X
Abnormal – walking aids used		X	X			
Wheelchair outdoors/long trips						
Mostly confined to a wheelchair						
Tendon reflexes						
Biceps	Normal	Normal	Diminished	Diminished	Normal	Normal
Triceps	Normal	Normal	Diminished	Diminished	Normal	Normal
Knee	Absent	Absent	Absent	Absent	Normal	Normal
Ankle	Absent	Absent	Absent	Absent	Diminished	Diminished
Sensory						
Joint position sense	Normal	Normal	Normal	Normal	Normal	Normal
Vibration	Reduced at elbow/knee	Reduced above elbow/knee	Reduced at elbow/knee	Reduced at elbow/knee	Reduced above elbow/knee	Reduced at elbow/knee
Pain/touch	Normal	Normal	Reduced in fingers/toes	Normal	Reduced in fingers/toes	Reduced in fingers/toes
Nerve conduction studies	Axonal motor and sensory neuropathy	Axonal motor and sensory neuropathy	Axonal motor and sensory neuropathy	Axonal motor and sensory neuropathy	Axonal predominantly motor neuropathy	Axonal motor neuropathy
Additional features						
Neuromyotonia	No	No	Yes	Yes	No	No
Pes cavus	No	Yes	Yes	No	Yes	Yes
Scoliosis	No	No	No	Yes	Yes	No
Tremor	Yes	Yes	No	Yes	Yes	Yes
Bradykinesia	No	No	No	No	Yes	No
Spasticity	No	No	No	No	No	No
Sensory ataxia	No	No	No	No	Yes	No
Deafness (hearing loss)	No	No	No	No	No	Yes
Cerebellar dysfunction	No	No	No	No	No	Yes
Bulbar dysfunction	No	No	No	No	No	No
Diaphragmatic dysfunction	No	No	No	No	No	No
Ulcerations	No	No	No	No	No	No
Nystagmus	No	No	No	Yes^b^	No	Yes^b^
Abnormal pupillary reaction	No	No	No	No	Yes^c^	Yes^c^
Other	No	No	No	No	No	No
CK level	93 U/L	273 U/L	749 U/L	^a^	^a^	^a^
CMTNS	10	15	9	12	12	14
Severity	Mild	Moderate	Mild	Moderate	Moderate	Moderate
ONLS						
Arms	1	1	2	2	2	2
Legs	3	4	2	3	4	4
Total	4	5	4	5	6	6

Abbreviations: CK, creatine kinese; CMTES, Charcot–Marie‐tooth examination score; CMTNS, Charcot–Marie‐tooth neuropathy score; ONLS, overall neuropathy limitations scale.

^a^ Not obtained.

^b^ Horizontal nystagmus.

^c^ Abnormal pupillary reaction to convergence.

Neuromyotonia on electromyography (P3, P4) and scoliosis (P4, P5) were present in two patients (P3 and P4/P 4 and P5, respectively). Pes cavus was observed in four patients (P2, P3, P5, P6). Postural tremor was observed in five (mild in P1, P4, P5, P6 and moderate in P2), and bradykinesia in one (P5). Sensory ataxia, hearing loss, and cerebellar dysfunction were present in single cases (P5, P6, respectively). Horizontal nystagmus was observed in two patients (P4, P6). Abnormal pupillary reactions were observed in two patients (P5, P6). Longitudinal data was available for three patients (P1, P2, P3) and all patients showed a progressive loss of motor function during follow up (6.5 years in P1 and P2; 1.5 years in P3). NCS revealed a length‐dependent axonal predominantly motor but not pure motor neuropathy in all patients (Table 4).

**TABLE 4:** Nerve conduction studies in patients with *HINT1*‐related polyneuropathy.

	Motor nerves								Sensory nerves					
	Median nerve		Ulnar nerve		Tibial nerve		Peroneal nerve		Median nerve		Ulnar nerve		Sural nerve	
	NCV, m/s	CMAP, mV	NCV, m/s	CMAP, mV	NCV, m/s	CMAP, mV	NCV, m/s	CMAP, mV	NCV, m/s	SNAP, μV	NCV, m/s	SNAP, μV	NCV, m/s	SNAP, μV
Patient 1	48.8	2.9	48.8	7.9	NA	NR	NA	NR	57.4	47.5	49.1	37.7	33.8	14.1
Patient 2	47.6	4.4	48.3	0.4	NA	NR	NA	NR	53.8	11.8	10.6	51.7	NA	NR
Patient 3	55.6	5.9	54.1	2.8	NA	NR	NA	NR	46.7	18.5	46.8	16.8	NA	NR
Patient 4	48.0	1.7	43.0	5.4	36.0	0.1	NA	NR	56	29.1	n.d.	n.d.	37.0	0.72
Patient 5	49.3	7.1	55.1	8.5	37.5	0.3	42.0	0.1	49.1	53.5	52.1	38.1	35.5	4.7
Patient 6	44.2	9.8	64.3	8.2	2.8	38.5	NA	NR	50.0	41.3	48.4	25.8	34.8	27.8

Abbreviations: CMAP, compound muscle action potential; NA, not applicable; NCV, nerve conduction velocity; n.d., not done; NR, no response to stimulation; SNAP, sensory nerve action potential.

### 3.3 Non‐Motor Symptoms and Quality of Life in Patients With *DNAJB2* ‐ and *HINT1* ‐Related Hereditary Neuropathy

**FIGURE 2:**
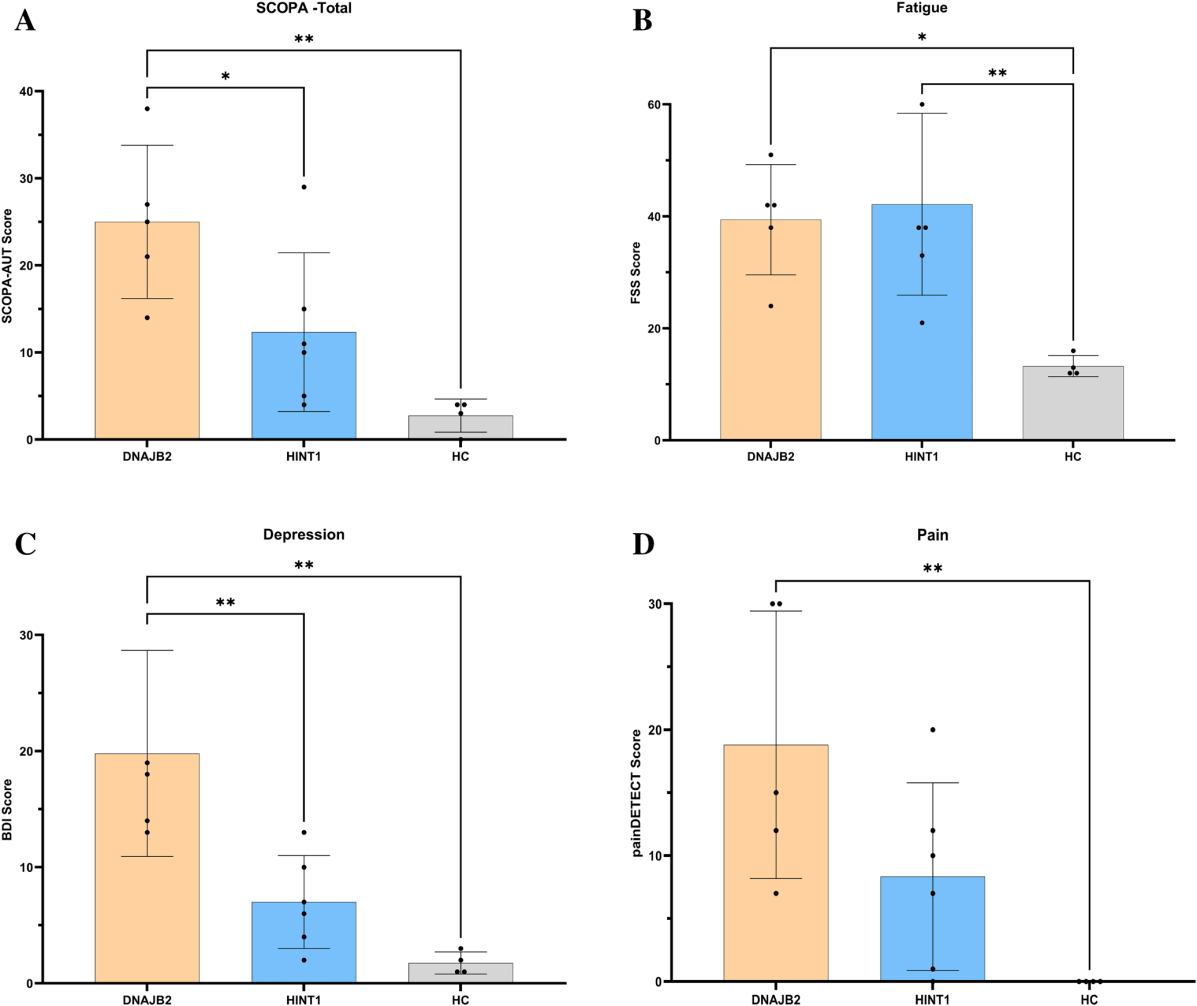
(A–D) Non‐motor symptoms in patients with *DNAJB2* and *HINT1* variants in comparison to HCs. BDI, beck depression inventory; *DNAJB2*, *Dnaj homolog subfamily B member 2*; FSS, fatigue severity scale; HC, healthy controls; *HINT1*, *histidine triad nucleotide‐binding protein 1*; SCOPA‐AUT, scale for outcomes in Parkinson's disease‐autonomic. Results were compared by one‐way ANOVA followed by Tukey's multiple comparison test ((A) F (2, 12) = 9194; *p* = 0.0038; (B) F (2, 12) = 7916; *p* = 0.0064; (C) F (2, 12) =12,17; *p* = 0.0013 and (D) F (2, 12) = 6604; *p* = 0.0116). All data are illustrated graphically as a box plot from min to max, (*p < 0.05; **p < 0.01); The figure displays means with standard deviations; circles represent individual patients (*n* = 4–6).

Figure 2A–D present the results of the SCOPA‐AUT, BDI, FSS, and painDETECT scores in patients with *DNAJB2*‐ and *HINT1*‐related neuropathies, compared to HCs. The median age at the time of testing was 33.5 years [range: 22.0–44.0] for patients with *DNAJB2* variants, 35.0 years [21.0–54.0] for patients with *HINT1* variants, and 33.0 years [23.0–37.0] for HC. Patients with *DNAJB2* variants showed significantly worse autonomic function measured with SCOPA‐AUT compared to patients with *HINT1* variants (25.0 [14.0–38.0] vs. 10.5 [4.0–29.0], *p* < 0.001) and healthy controls (HC) (25.0 [14.0–38.0] vs. 3.5 [0.0–4.0], *p* < 0.001) (Figure 2A). Patients with *DNAJB2* variants also showed worse fatigue measured with FSS than HCs (42.0 [24.0–51.0] vs. 12.5 [12.0–16.0], *p* = 0.02) (Figure 2B), worse depression measured with BDI than patients with *HINT1* variants (18.0 [13.0–35.0] vs. 6.5 [1.0–13.0], *p* = 0.008) and HCs (18.0 [13.0–35.0] vs. 1.5 [1.0–3.0], *p* = 0.002) (Figure 2C), and higher painDETECT scores than HCs (15.0 [7.0–30.0] vs. 0.0 [0.0–0.0], *p* = 0.009) (Figure 2D). In contrast, patients with *HINT1* variants showed significantly higher scores only for the FSS compared with HC (38.0 [21.0–63.0] vs. 12.5 [12.0–16.0], *p* = 0.007) (Figure 2B). Neuropathic pain was present in two of five patients with *DNAJB2* variants (P4 and P5) and one of six patients with *HINT1* variants (P6). All patients with *DNAJB2* variants met criteria for depression, while only one patient with *HINT1* variants had depression (P4). Significant fatigue was reported in four of five patients with *DNAJB2* variants (P1, P2, P3, P4) and four of six patients with *HINT1* variants (P2, P3, P5, P6). RLS was noted in four of five patients with *DNAJB2* variants (P2, P3, P4, P5; median IRLS‐SS score: 29.0 [15.0–36.0]), but in none of the patients with *HINT1* variants. Among the affected individuals with *DNAJB2* variants, one had moderate (P3), one had severe (P2), and two had very severe RLS (P4, P5).

**FIGURE 3:**
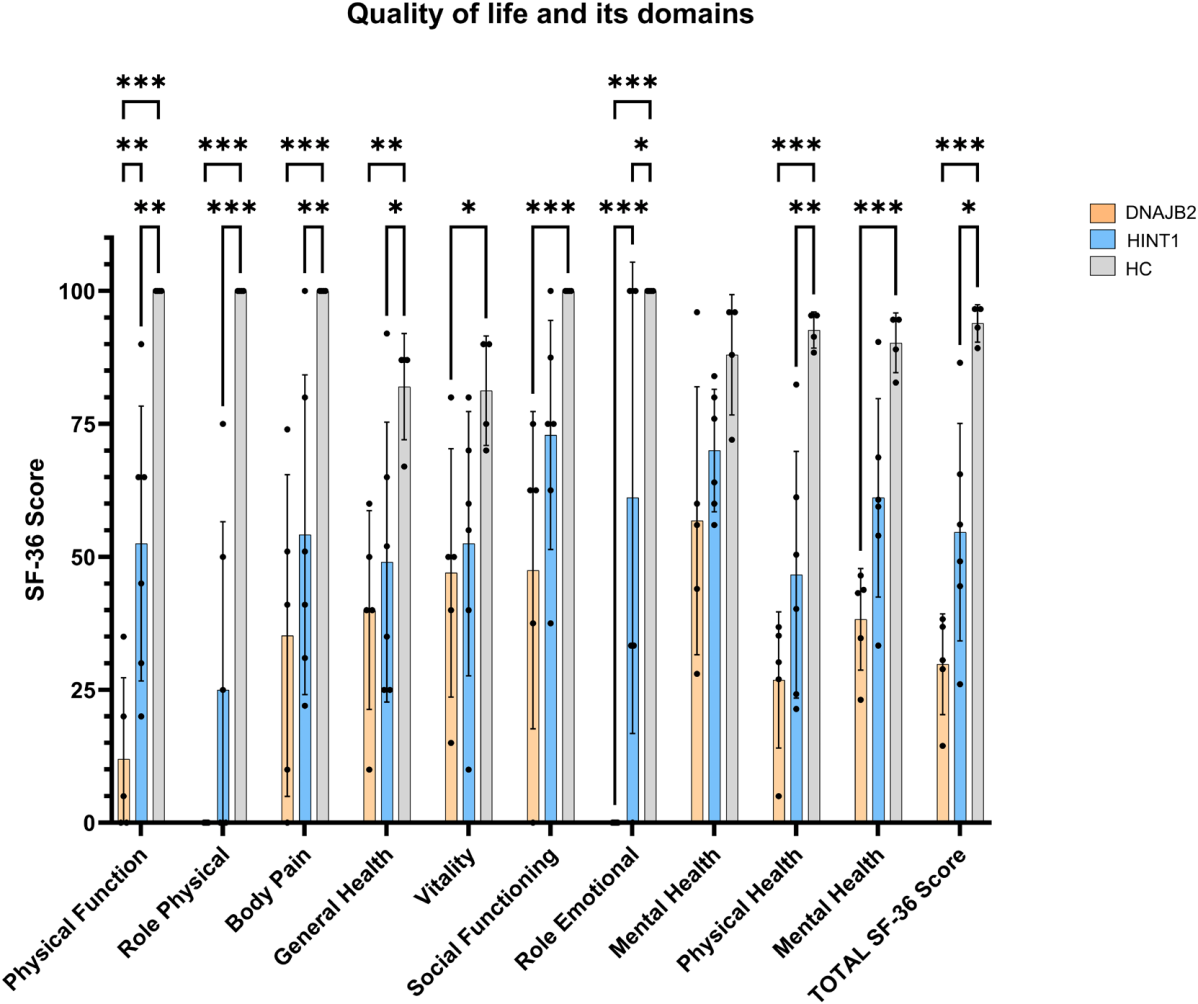
Quality of life and its domains in patients with *DNAJB2* and *HINT1* variants in comparison to HC. *DNAJB2*, *Dnaj homolog subfamily B member 2*; HC, healthy controls; *HINT1*, *histidine triad nucleotide‐binding protein 1*; SF‐36, short form‐36; The figure displays means with standard deviations; circles, triangles, and crosses represent individual patients (*n* = 4–6). Results were compared by two‐way ANOVA followed by Tukey's Multiple Comparison Test (F(2,132) = 2,66; *p* = 0.0055; All data are illustrated graphically as a box plot from min to max, *p < 0.05; **p < 0.01; ***p < 0.001). The figure displays means with standard deviations; circles represent individual patients (*n* = 4–6).

Figure 3 shows the results of the SF‐36 total score and its domains in patients with *DNAJB2*‐ and *HINT1*‐related neuropathies compared with healthy controls (HCs). The most affected QoL domains in patients with *DNAJB2* variants were RP and RE, where they scored the lowest possible score (zero). The total SF‐36 score was significantly lower in patients with *DNAJB2* variants compared with HC (30.6 [14.4–38.3] vs. 94.9 [89.2–96.6], *p* < 0.001). Patients with *HINT1* variant also had a significantly lower total SF‐36 score compared with HC (52.6 [26.1–86.5] vs. 94.9 [89.2–96.6], *p* = 0.01). There was no statistically significant difference in total SF‐36 score between patients with *DNAJB2* variants and patients with *HINT1* variants.

Disease severity (CMTNS) showed a strong negative correlation with QoL (total SF‐36 score; rho = −0.94), as well as a moderate positive correlation with fatigue severity (FSS score; rho = 0.61) and pain severity (painDETECT score; rho = 0.58) in patients with *HINT1* variants. No correlations were found in patients with *DNAJB2* variants.

## 4 Discussion

Differentiating hereditary axonal polyneuropathies caused by distinct gene variants remains a clinical challenge. Here, we compared age‐matched patients carrying two rare variants associated with axonal polyneuropathy—*HINT1* and *DNAJB2*. Beyond the motor phenotype, we systematically evaluated non‐motor manifestations, including autonomic dysfunction, fatigue, RLS, and neuropathic pain, as well as QoL. Furthermore, we report novel variants in the *DNAJB2* gene and explore its genotype–phenotype correlation.

Both groups of patients with *DNAJB2*‐ and *HINT1* neuropathy exhibited a CMT2 phenotype. However, NCS more commonly showed pure motor involvement in patients with *DNAJB2* variants. Disease onset was typically later in patients with *DNAJB2* variants (late adolescence or early adulthood) compared to patients with *HINT1* variants (childhood or early adolescence), yet patients with *DNAJB2* variants more frequently developed severe neuropathy, as reflected by higher CMTNS scores. The spectrum of additional clinical features also differed between the two groups. All patients with *DNAJB2* variants presented with pes cavus, and five out of six had scoliosis, likely reflecting the severity of neuropathy. In contrast, five out of six patients with *HINT1* pathogenic variants exhibited a postural tremor, and one patient additionally showed bradykinesia. So far, tremor (irregular tremor on the finger–nose test) has only been reported in a single patient with *HINT1* variant from France [28]. A Parkinson's disease (PD) phenotype has previously been described as part of the clinical spectrum of *DNAJB2*‐related neuropathy [5, 10, 11, 29]. In our cohort, none of the patients with *DNAJB2* variants had a full PD phenotype, although two patients showed mild resting and postural tremor. Furthermore, four out of five patients with *DNAJB2* variants but none of the patients with *HINT1* variants had RLS, a condition known to be associated with PD [30]. This finding further supports a possible association between *DNAJB2* variants and the clinical spectrum of PD. Mild bulbar dysfunction was observed in two patients with *DNAJB2* variants and in none of the patients with *HINT1* variants. Frasquet and colleagues reported severe bulbar involvement and the need for percutaneous endoscopic gastrostomy (PEG) in three patients with homozygous *DNAJB2* c.352 + 1G>A variants, but this occurred in the later stages of disease (at ages 74, 60, and 63 years). In contrast, our patients exhibited mild bulbar symptoms much earlier, at ages 20 and 21. Mild hearing loss was present only in one patient with *DNAJB2* variant. Saveri and colleagues described an Italian family with three patients carrying a homozygous pathogenic *DNAJB2* variant (c.145delG), all of whom had hearing loss. Our patient with *DNAJB2* variant with hearing loss was the oldest in the cohort (44 years) and had severe neuropathy. This observation is consistent with the report by Saveri et al., in which all affected individuals were older (52, 59, and 61 years) and also exhibited severe neuropathy. In *HINT1*‐related neuropathy, hearing loss has not been reported to date. Finally, neuromyotonia on electromyography, a diagnostic hallmark of *HINT1* neuropathy [13], was not present in any of our patients with *DNAJB2* variants but in two patients with *HINT1* variants, as expected.

This study provides longitudinal data on motor function in patients with *DNAJB2* variants. We observed that all patients with *DNAJB2* variants experienced a progressive loss of motor function over a follow‐up period of up to 10 years, with an annual decline in the MRC‐SS score of 0.78 points per year. In comparison, Shy et al. reported an annual rate of change in motor function of 0.061 for the arms and 0.118 for the legs in patients with CMT1A [31]. However, their analysis was based on subdivided motor scores from the CMTNS, rather than the MRC‐SS score used in our study so that they cannot be directly compared.

We examined, to the best of our knowledge, for the first time, the presence of autonomic dysfunction, fatigue, depression, neuropathic pain, and QoL in patients with *DNAJB2*‐ and *HINT1*‐related neuropathies. Patients with *DNAJB2* variants scored worse than HC across all evaluated measures and additionally showed greater autonomic dysfunction and depression severity compared with patients with *HINT1* variants. A moderate positive correlation between disease severity and fatigue severity as well as pain severity was observed only in patients with *HINT1* variants. However, the statistical analysis should be interpreted with caution due to the very small cohort size. In contrast to our results, a large Italian registry study including 251 CMT patients reported that 36% of patients had abnormal fatigue (compared with 80% and 66% in our patients with *DNAJB2* and *HINT1* variants, respectively), assessed using the Modified Fatigue Impact Scale (MFIS) [32]. Bellofatto et al. also identified significant correlations between fatigue and disease severity, anxiety/depression/general distress, somnolence, obesity (BMI ≥ 30), and the use of anxiolytic/antidepressant or anti‐inflammatory/analgesic medications [33]. Neuropathic pain was reported in approximately 30% of Serbian CMT1A patients, which is somewhat comparable to our findings (40% in patients with *DNAJB2* variants and 16% in those with *HINT1* variants), taking into account the small sample size of the present study [34].

Furthermore, overall QoL was significantly reduced in patients with *DNAJB2* variants compared with HC, with role physical and role emotional domains being the most affected domains. These two domains also differed significantly between patients with *DNAJB2* and *HINT1* variants (Figure 3), with patients with *DNAJB2* variants having worse scores. A probable explanation for this discrepancy may be that patients with *HINT1* variants, who experienced first symptoms earlier in life, may have developed compensatory mechanisms that allow them to cope more effectively with the disease. When compared with Serbian patients with CMT1A, patients with *HINT1* variants demonstrated a very similar overall QoL [35]. More pronounced multisystemic involvement in patients with *DNAJB2* variants may further impact their QoL. Another important factor that may have contributed to the reduced QoL in the patients with *DNAJB2* variants in our cohort is the fact that they were refugees from Syria and Iran resettled in Germany, as the association between pre‐, peri‐, and post‐migration stressors and mental ill health in refugees in high‐income host countries is well documented [36, 37, 38]. This is in line with our findings, as all of our patients with *DNAJB2* variants met the criteria for depression. In the study by Bellofatto et al., which included 252 CMT patients with various subtypes, 10% of patients demonstrated moderate‐to‐severe depression (assessed with the Hospital Anxiety and Depression Scale). This proportion is comparable to the prevalence of depression in our *HINT1* patient group (16%). Cordeiro et al. concluded in a systematic review of 20 studies that CMT patients show an increased tendency toward depressive symptoms compared with the general population and are at higher risk of reduced QoL [39].

While our study has several strengths, including follow‐up of motor function in patients with *DNAJB2* variants and the first characterization of non‐motor symptoms and QoL in these patients, the main limitations are the small sample size due to the rarity of the disease and the lack of functional analyses of the pathogenic variants. In addition, psychiatric and cognitive features were not systematically assessed in patients with *HINT1* variants, representing another limitation, given emerging evidence suggesting a potential role of HINT1 in psychiatric disorders [40, 41].

In conclusion, we expand the clinical spectrum of *DNAJB2*‐ and *HINT1*‐related hereditary neuropathies, describing the phenotype beyond motor symptoms and providing a direct comparison of these two rare axonal neuropathies.

## Funding

Hannover Medical School and Deutsche Forschungsgemeinschaft (DFG ME 3696/3).

## Conflicts of Interest

B.B. received compensation for travel expenses from ITF Pharma GmbH, the German Neuromuscular Society “Deutsche Gesellschaft fuer Muskelkranke” (DGM e.V.) and German Society for Clinical Neurophysiology and Functional Imaging (“Deutsche Gesellschaft für Klinische Neurophysiologie und Funktionelle Bildgebung” – DGKN e.V.) and served on advisory boards of Roche all outside of the submitted work. A.H. has received honoraria as a speaker/consultant from Alexion, BMS, Galapagos, Kyverna, Medscape, Merck Serono, Neuraxpharm, Roche, Sandos, Sanofi outside of the submitted work. S.P. has received speaker fees, non‐financial support and research support from Biogen, Roche, AL‐S Pharma, Amylyx, Cytokinetics, Ferrer, ITF‐Pharma, and Sanofi and served on advisory boards of Amylyx, Biogen, Roche, Zambon and ITF Pharma outside of the submitted work. C.H., S.H., M.V., S.K., T.G. and S.P. declare no conflicts of interest.

## Data Availability

The data that support the findings of this study are available from the corresponding author upon reasonable request.
